# Indoxyl sulfate is associated with mortality after AKI – more evidence needed!

**DOI:** 10.1186/s12882-019-1465-0

**Published:** 2019-07-26

**Authors:** Steven Menez, Mohamad Hanouneh, Tariq Shafi, Bernard G. Jaar

**Affiliations:** 1 Division of Nephrology, Department of Medicine, Johns Hopkins University School of Medicine, 1830 E. Monument Street, Suite 416, Baltimore, MD 21287 USA; 2 Department of Epidemiology, Johns Hopkins Bloomberg School of Public Health, 615 N. Wolfe Street, Baltimore, MD 21205 USA; 3 Welch Center for Prevention, Epidemiology and Clinical Research, Johns Hopkins University, 2024 E. Monument Street, Baltimore, MD 21205 USA; 4 Nephrology Center of Maryland, 5601 Loch Raven Boulevard, Suite 3 North, Baltimore, MD 21239 USA; 5 Division of Nephrology, Department of Medicine, University of Mississippi Medical Center, Jackson, MS USA

**Keywords:** AKI, Mortality, Risk factors, Indoxyl sulfate

## Abstract

Patients who develop acute kidney injury (AKI) have significantly higher short-term outcomes including in-hospital mortality. The development of AKI has been associated with long-term consequences including progression to chronic kidney disease (CKD) and higher rates of cardiovascular disease (CVD) and mortality. In recent years there has been a growing push for the discovery of novel methods to diagnose AKI at earlier stages, and for an improvement in risk stratification and prognosis following AKI.

Wang and colleagues assessed the association of total serum indoxyl sulfate (IS) levels, a protein bound uremic toxin, with 90-day mortality after hospital-acquired AKI (HA-AKI). These authors found that serum IS levels were significantly elevated in patients with HA-AKI (2.74 ± 0.75 μg/mL) compared to healthy subjects (1.73 ± 0.11 μg/ml, *P* < 0.001) and critically ill patients (2.46 ± 0.35 μg/ml, *P* = 0.016).

The mechanisms of this relationship remain unclear, with a limited understanding of cause-specific mortality associated with either the high or low-IS group. One limitation of this current study is an understanding of the acceptable or expected higher level in IS during episodes of AKI. IS levels remained persistently elevated at day 7 compared to β2-microglobulin and serum creatinine which were both lower at 7 days. It is unclear, however, if levels of β2-microglobulin and serum creatinine were lower for other reasons, such as if any patients with AKI required dialysis.

This work provides an important addition to the field of AKI research, specifically in the evaluation of readily measurable biomarkers and outcomes after AKI. Moving forward, further validation in studies of acute kidney injury are needed to develop a better understanding of IS levels at the time of AKI diagnosis and trends during the course of AKI.

## Commentary

Acute kidney injury (AKI) is a major public health burden that affects millions of people globally every year [1–3]. Among hospitalized patients, various studies have reported an annual incidence of AKI ranging between 20 and 25%, with rates exceeding 50% in the intensive care unit [4–6]. Patients with AKI have significantly higher in-hospital mortality, with increased resource utilization, compared to hospitalized patients who do not experience AKI [1, 7]. Further, the development of AKI has been associated with long-term consequences including progression to chronic kidney disease (CKD), higher rates of cardiovascular disease (CVD), and increased mortality [8–10].

It is widely recognized that standard methods used to diagnose AKI, including serum creatinine and urine output, can potentially lead to a delay in diagnosis and sometimes misdiagnosis of AKI [11, 12]. Therefore, in recent years there has been a growing push for the discovery of novel methods to diagnose AKI at earlier stages and improve both risk stratification and prognosis following AKI. Consequently, there has been a significant increase in the evaluation of novel urinary or blood biomarkers of kidney injury during the period of, and following episodes of AKI [13–15].

**Fig. 1:**
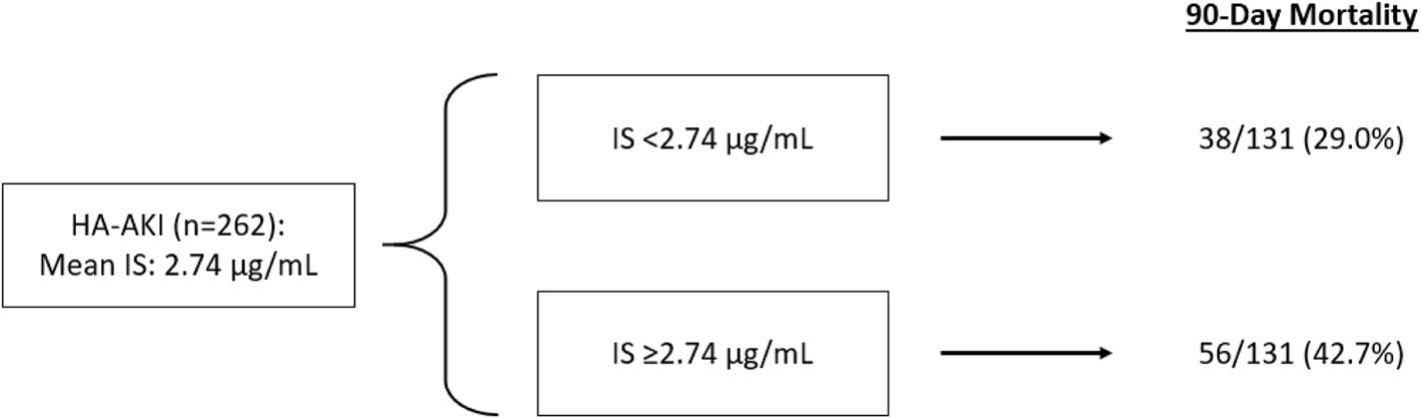
Association between IS level at the time of AKI diagnosis and 90-day mortality. * HA-AKI: hospital-acquired acute kidney injury. * IS: indoxyl sulfate

Wang et al. previously evaluated the association between serum pre-albumin levels with outcomes after hospital-acquired AKI [16]. In their prior study using the same population cohort, the authors noted that relatively lower serum pre-albumin levels at the time of AKI diagnosis, as well as a relatively larger change in pre-albumin level over the course of 7 days, was associated with a significantly increased risk of 90-day mortality. In this article, Wang and colleagues assessed the association of total serum indoxyl sulfate (IS) levels, a protein bound uremic toxin, with 90-day mortality after hospital-acquired AKI (Fig. 1).

One major strength of this current study is the choice of IS, a protein-bound uremic toxin, and its association with 90-day mortality, given the biological plausibility of its role in the development of, and clinical course after, AKI noted in several studies. Protein-bound uremic toxins have been shown in patients with CKD to be associated with worse outcomes [17]. As the authors describe, IS is an end product of metabolism of dietary tryptophan, levels of which can be significantly elevated in the setting of CKD [18]. IS may play a central role in inflammation and endothelial cell injury in the setting of AKI [19, 20]. Murine studies have elucidated the role of increasing IS levels with a subsequent rise in inflammatory markers such as Interleukin 1 beta (IL-1β), phosphorylation of Mitogen-Activated Protein Kinase (MAPK), and activation of *Nuclear Factor* kappa-light-chain-enhancer of activated B cells (NF-κB) and *Activator Protein 1* (AP-1). IS may prime endothelial cells for inflammation, with a second hit leading to endothelial cell injury, ultimately resulting in kidney injury [19].

However, it is still unclear whether IS serves as a *direct* toxin or could be an indicator, instead, of other unknown toxins. An elevated serum level of IS may itself be due to impaired clearance of this solute by tubular secretion in the setting of tubular injury and downregulation of organic anion transporters (OATs), but the association between IS level and clinical outcomes is certainly more complex. In the setting of sepsis in particular, other factors may lead to a higher production of IS such as changes in a patient’s microbiome from antibiotics [21]. Prior research has shown that short-term mortality following AKI can be due to factors not directly related to AKI such as older age, other organ dysfunction, and coagulopathy [22, 23].

One limitation of this current study is an understanding of the acceptable or expected elevation in IS during episodes of AKI. For their primary analyses the authors used the mean value of 2.74 μg/mL in patients with AKI, significantly higher than the mean level in healthy subjects (1.73 μg/mL; *p* < 0.001). The authors appropriately note that this was higher than in critically ill patients without AKI (2.46 μg/mL; *p* = 0.02) but significantly *lower* than in patients with CKD (3.07 μg/mL, *p* < 0.001). In their Cox regression models, the authors analyze IS dichotomously as well as continuously and show a significantly increased risk of 90-day mortality with higher levels. While the authors adjust for many potential confounders in their modeling, it would be worth evaluating the how these associations with IS and mortality compare against a more precise measure of kidney function, such as dynamic GFR in the setting of AKI [24].

Another area of uncertainty in this study is the course of the patients during and after hospitalization. The authors argue that IS levels remained persistently elevated at day 7 in 89 patients in their cohort, compared to β2-microglobulin and serum creatinine which were both lower at 7 days, and that overall elevated IS levels may more accurately reflect kidney injury. It is unclear, however, if levels of β2-microglobulin and serum creatinine were lower for other reasons, such as if any patients with AKI required dialysis. The authors rightly point out in their limitations that there are no data regarding urine output and cause of death. While a higher overall mortality in patients with elevated IS level following AKI was clearly observed, greater clarification into the cause of death is also warranted.

This work provides an important addition to the field of AKI research, specifically in the evaluation of readily measurable biomarkers and outcomes after AKI. As the authors have shown, the use of IS was more clearly associated with mortality than either serum creatinine or β2-microglobulin. Moving forward, further validation in studies of AKI are needed to develop a better understanding of IS levels at the time of AKI diagnosis and trends during the course of AKI. Ultimately, however, the question of utility must be addressed prior to any clinical implementation. Previously, the association between IS and inflammation in the CKD population led to increased attention as a potential therapeutic target in slowing CKD progression [25, 26]. However, despite research showing an effective reduction in IS levels with drugs such as AST-120 in patients with CKD and ESRD, no benefit has been demonstrated in the use of AST-120 with all-cause and cardiovascular mortality [27–29]. While these data are not convincing in the CKD population, reduction of IS in the setting of AKI has yet to be explored.

In summary, Wang and colleagues describe the association between elevated IS level and 90-day mortality after hospital-acquired AKI. The mechanisms of this relationship remain unclear however, with a limited understanding of cause-specific mortality associated with either the high or low-IS group. Further, other major cardiovascular and kidney-related outcomes were not explored but warrant additional investigation. Findings of this study will need to be validated and if confirmed, the use of IS may hold significant potential for clinical use..

## Data Availability

N/A

## Abbreviations

AKI: Acute kidney injury
AP-1: *Activator protein 1*
CKD: Chronic kidney disease
CVD: Cardiovascular disease
ESKD: End stage kidney disease
IL-1β: Interleukin 1 beta
IS: Indoxyl sulfate
MAPK: Mitogen-activated protein kinase
NF-κB: *Nuclear factor* kappa-light-chain-enhancer of activated B cells
OAT: Organic anion transporter
